# Frameless stereotaxy in subthalamic deep brain stimulation: 3-year clinical outcome

**DOI:** 10.1007/s10072-020-04561-9

**Published:** 2020-07-07

**Authors:** Carla Piano, Francesco Bove, Delia Mulas, Anna Rita Bentivoglio, Beatrice Cioni, Tommaso Tufo

**Affiliations:** 1 Institute of Neurology, Fondazione Policlinico Universitario A. Gemelli IRCCS, Università Cattolica del Sacro Cuore, Largo A. Gemelli 8, 00168 Rome, Italy; 2 Institute of Neurology, Mater Olbia Hospital, Olbia, Italy; 3 Institute of Neurosurgery, Fondazione Policlinico Universitario A. Gemelli IRCCS, Università Cattolica del Sacro Cuore, Rome, Italy

**Keywords:** Deep brain stimulation, Frameless, Subthalamic nucleus, Parkinson’s disease, Personalized medicine

## Abstract

**Background:**

In most centers, the surgery of deep brain stimulation (DBS) is performed using a stereotactic frame. Compared with frame-based technique, frameless stereotaxy reduces the duration of surgical procedure and patient’s discomfort, with lead placing accuracy equivalent after the learning curve. Although several studies have investigated the targeting accuracy of this technique, only a few studies reported clinical outcomes, with data of short-term follow-up.

**Objective:**

To assess clinical efficacy and safety of frameless bilateral subthalamic nucleus (STN) DBS in Parkinson’s disease (PD) patients at 1- and 3-year follow-up.

**Methods:**

Consecutive PD patients who underwent bilateral STN-DBS with a manual adjustable frameless system were included in the study. The data were collected retrospectively.

**Results:**

Eighteen PD patients underwent bilateral STN-DBS implant and were included in the study. All patients completed 1-year observation and ten of them completed 3-year observation. At 1-year follow-up, motor efficacy of STN stimulation in off-med condition was of 30.1% (*P* = 0.003) and at 3-year follow-up was of 36.3%, compared with off-stim condition at 3-year follow-up (*P* = 0.005). Dopaminergic drugs were significantly reduced by 31.2% 1 year after the intervention (*P* = 0.003) and 31.7% 3 years after the intervention (*P* = 0.04). No serious adverse events occurred during surgery.

**Conclusions:**

Frameless stereotaxy is an effective and safe technique for DBS surgery at 1- and 3-year follow-up, with great advantages for patients’ discomfort during surgery.

## Introduction

In advanced Parkinson’s disease (PD), there is strong evidence that deep brain stimulation (DBS) of the subthalamic nucleus (STN) outperforms best medical treatment in controlling motor symptoms and drug-induced complications and improving quality of life in short- and long-term follow-up [1, 2]. Accurately placing of leads into the targeted deep nucleus is an essential step for successful DBS therapy [3]. Misplacement of leads is one of the most common causes of DBS failure, resulting in unsatisfactory improvement and/or adverse stimulation effects [4]. In order to minimize failures and side effects, a full multidisciplinary preoperative assessment is mandatory; moreover, the neurosurgeon should take into consideration possible variables, such as brain shift during stereotactic implantation [5].

In most centers, the procedure is performed using a stereotactic frame where the patient’s skull is fixed [6]. During the surgical procedure, the reference frame can be coupled with a CT/MRI visible adapter, depending on the imaging modality used for targeting and for registering the images to patient’s head [7]. These systems also require a CT or MRI scan to be taken immediately after the placement of frame. The process of frame placement, image acquisition, calculation, and then translation of the coordinates for the frame can take a significant amount of time, and not only increases patients’ discomfort but also use valuable operative room resources and time. Furthermore, frame placement on patients awake who are off their medications can be challenging, and patients may have difficulty to tolerate the frame during a procedure that severely limits range of motion, lasts several hours, and requires active participation [8].

Frameless stereotaxy reduces the duration of surgical procedure, as imaging can be performed well in advance of surgery, avoiding a preoperative imaging after that the frame has been fixed, and reduces patients’ discomfort, because they are able to move their heads and reposition themselves during surgery. This is a disposable and sterile system, which does not need periodic recalibration of frame and arc. It also provides the possibility to target posterior areas of the brain without the need of inverting the frame, and minor adjustments can be made in multiple planes to the entry point without adjustment of frame and coordinates. Moreover, without the frame, intraoperative examination of the patient and eventual access to the airway are easier [9].

Some studies reported that frameless technique slightly lessened lead placing accuracy as compared with frame-based technique, without influencing clinical results of DBS [10, 11]. Conversely, other studies did not found significant differences between frameless and frame-based technique in lead placement accuracy [12–14]. These inconsistencies may be explained, at least partially, by different procedural experience in various centers, which influence the targeting accuracy. In fact, the learning curve of frameless procedure shows a twofold improvement of accuracy with increasing experience of the surgical team. Notably, after the first procedures, frameless accuracy is comparable with frame-based system accuracy [11].

Although several studies have investigated the targeting accuracy of frameless stereotaxy [9], only a few studies reported clinical outcomes, with data of short-term follow-up [15, 16]. In this retrospective study, we report 1- and 3-year clinical follow-up of PD patients who underwent STN-DBS with a manual adjustable frameless system.

## Methods

### Patients’ selection and data collection

Consecutive PD patients who underwent bilateral STN-DBS at a single tertiary care university hospital between 2012 and 2017 were included in the study. All patients had a diagnosis of Parkinson’s disease according to the UK Parkinson’s Disease Brain Bank criteria [17], and fulfilled the inclusion and exclusion criteria proposed by the core assessment program for surgical interventional therapies in Parkinson’s disease panel [18]. Patients with previous neurosurgical interventions for PD or implantation of DBS electrodes in other deep brain nuclei were excluded from the study. The data were collected from patients’ chart retrospectively.

### Surgical procedure

For each patient, contrast-enhanced volumetric T1-weighted 1.5 Tesla magnetic resonance imaging (MRI) of the whole head and T2-weighted images through the STN were obtained about 2 weeks before the procedure. The day before surgery, NexFrame Unibody bone fiducial markers (Medtronic, Inc., Minneapolis, MN) were applied to the skull after application of local anesthetic. A full-head computed tomography (CT) scan of 1-mm slice thickness was obtained throughout the cranial volume and all image data sets were loaded into the Medtronic StealthStation FrameLink software package. MRI and CT images were fused and the STN was targeted. The subthalamic nucleus was identified on T2 imaging and a scalable Schaltenbrand atlas was overlaid to aid in identification of the nuclear boundaries. Both direct and indirect methods were used to approximate the appropriate target location within the STN, with final targeting refined by microelectrode recording. Target coordinates were 11 to 12 mm lateral to the anterior commissure-posterior commissure (AC-PC) line, 3 to 5 mm below the AC-PC plane, and 2 to 3 mm posterior to the midpoint of AC-PC.

On the day of surgery, the patient was secured in position by a head cradle and cervical collar. A nonsterile reference arc was attached to the patient’s head and each fiducial marker was touched with a passive planar registration probe equipped with reflective spheres, which could be tracked by the cameras of the StealthStation. Entry points were marked on the scalp and the patient was prepared and draped in a sterile fashion. After burr-hole placement, the Medtronic Stimloc burr-hole cover was attached, followed by a Nexframe platform. A sterile reference arc was fastened to the base of the platform. Each fiducial marker was touched through the drape to perform registration again. Alignment of the trajectory was accomplished by adjusting the Nexframe platform to orient the trajectory to the planned target, using the guidance view of the target provided by the FrameLink software. The sensorimotor region of the STN was identified with single-track multipass microelectrode recording using 1 megohm platinum-iridium microelectrodes (FHC Corp, Bowdoinham, Maine). The number of tracks performed and track placement depended on the electrophysiological findings from the previous track. Unit recordings were performed with a Leadpoint 4 system (Medtronic, Minneapolis, MN). Intraoperative microstimulations were used to assess therapeutic effect and side effect thresholds [19]. After target location was identified, the DBS lead (model 3389, Medtronic, Minneapolis, MN) was implanted. The implantable pulse generator placement was performed under general anesthesia after lead placement. Postoperative CT scans were obtained to rule out hemorrhage and to verify lead location.

### Outcomes measurements

The objective of this study was to assess clinical efficacy and safety of frameless bilateral STN-DBS at 1- and 3-year follow-up.

The following variables were assessed at baseline (preoperatively), 1 year, and 3 years after the surgery:

- Score of the Unified Parkinson’s Disease Rating Scale (UPDRS) III and axial subscore (items 27–31) in off-medication (off-med) and on-med preoperatively, in off-med off-stimulation (off-stim), off-med on-stim, on-med off-stim, on-med on-stim postoperatively [20]
- Levodopa equivalent daily dose (LEDD) [21]

Adverse events related to stimulation or device were systematically collected at 1- and 3-year follow-up.

### Statistical analysis

Continuous data comparing baseline and postoperative scores at 1- and 3-year follow-up were analyzed by means of the Wilcoxon signed-rank test. Statistical significance was set at *P* < 0.05. All statistical computations were 2-sided and relied on Statistica 7.0 software (StatSoft, Tulsa, OK, USA).

## Results

### Demographic and clinical details of the patients included in the study

**Fig. 1:**
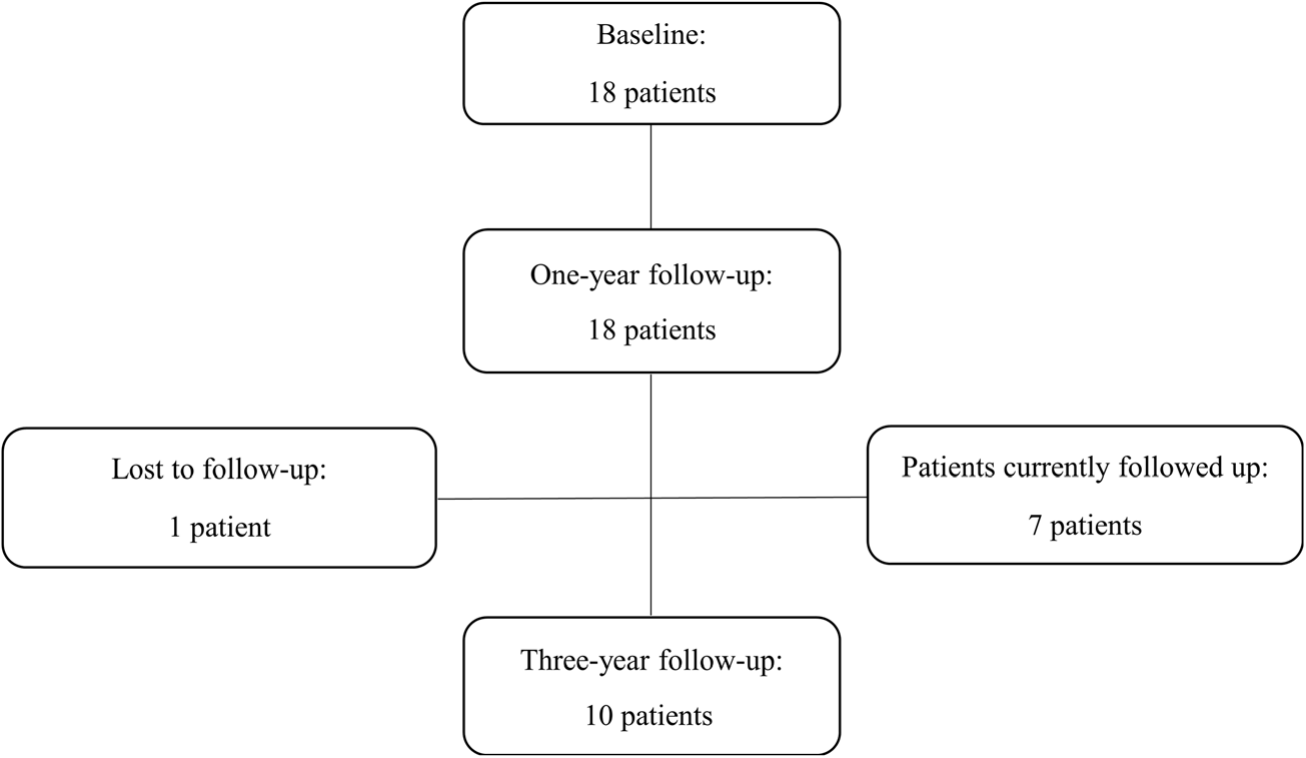
Study flow

Eighteen PD patients were included in the study (Table 1). Mean age at implantation was 55.6 ± 7.9 years and mean disease duration was 11.9 ± 6.2 years. MER tracks were performed per side and in 34 of 36 (94%) sides, the first track entered the sensorimotor region of STN, and no other MER tracks were performed. In each of the other two sides, two MER tracks were performed, and the lead was placed through the medial track. All patients completed 1-year observation and ten of them completed 3-year observation. Of the other eight patients, one was lost at follow-up and seven are currently being followed up (Fig. 1).

**Table 1:** Patients’ demographic and clinical data at baseline

	Total sample	Patients completing the 3-year period
Number	18	10
Gender (male/female)	11/7	5/5
Age at intervention (years)	55.6 ± 7.9	55.0 ± 9.2
Disease duration (years)	11.9 ± 6.2	10.5 ± 3.6
Hoehn and Yahr stage	2.8 ± 0.6	2.8 ± 0.8
UPDRS III off-med	30.2 ± 8.4	29.8 ± 10.2
UPDRS III on-med	17.4 ± 5.9	16.8 ± 6.5
LEDD (mg)	1117.2 ± 453.3	1111.2 ± 505.9

*Abbreviations*: *UPDRS*, Unified Parkinson’s Disease Rating Scale; *med*, medications; *LEDD*, levodopa equivalent daily dose

Data are shown as mean ± standard deviation

### Efficacy of frameless STN-DBS at 1- and 3-year follow-up

**Fig. 2:**
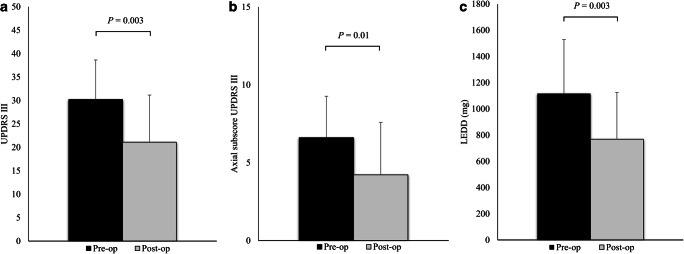
One-year follow-up of the 18 STN-DBS subjects. Data are presented as mean and standard deviation. **a** Preoperative off-medication versus postoperative off-medication on-stimulation UPDRS III scores. **b** Preoperative off-medication versus postoperative off-medication on-stimulation UPDRS III axial subscores. **c** Preoperative versus postoperative levodopa equivalent doses (LEDD)

At 1-year follow-up (Fig. 2), motor efficacy of STN stimulation in off-med condition was of 30.1% (preoperative UPDRS III off-med 30.2 ± 8.4 versus postoperative UPDRS III off-med on-stim 21.1 ± 10.0, *P* = 0.003). The benefit was significant also considering the axial symptoms, with 36.4% of improvement of UPDRS III axial subscore (preoperative axial subscore off-med 6.6 ± 2.7 versus postoperative axial subscore off-med on-stim 4.2 ± 3.4, *P* = 0.01). Likewise, dopaminergic drugs were significantly reduced by 31.2% 1 year after the intervention (mean preoperative LEDD 1117.2 ± 453.3 mg versus mean postoperative LEDD 768.8 ± 412.9 mg, *P* = 0.003).

**Fig. 3:**
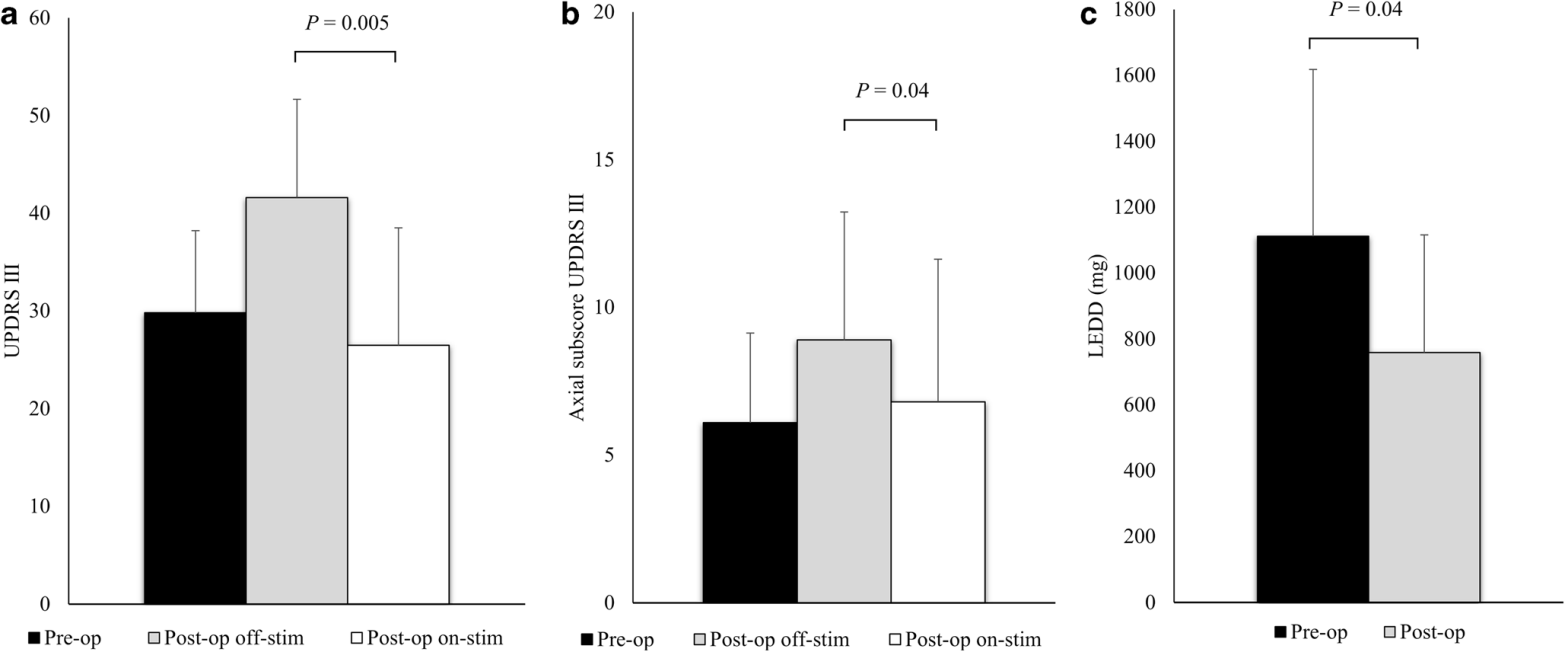
Three-year follow-up of the 10 STN-DBS subjects. Data are presented as mean and standard deviation. **a** Preoperative off-medication and postoperative off-medication off-stimulation versus postoperative off-medication on-stimulation UPDRS III scores. **b** Preoperative off-medication and postoperative off-medication off-stimulation versus postoperative off-medication on-stimulation UPDRS III axial subscores. **c** Preoperative versus postoperative levodopa equivalent doses (LEDD)

At 3 -year follow-up (Fig. 3), ten patients completed the observation. Motor efficacy of STN stimulation in off-med condition was 11.1% compared with preoperative condition (preoperative UPDRS III off-med 29.8 ± 10.2 versus postoperative UPDRS III off-med on-stim 26.5 ± 12.0, *P* = 0.3) and 36.3% compared with off-stim condition at 3-year follow-up (postoperative UPDRS III off-med off-stim 41.6 ± 14.1 versus postoperative UPDRS III off-med on-stim 26.5 ± 12.0, *P* = 0.005). Axial symptoms were not improved compared with preoperative condition (preoperative axial subscore off-med 6.1 ± 3.0 versus postoperative axial subscore off-med on-stim 6.8 ± 4.8, *P* = 0.5), but significantly improved of 23.6% compared with off-med off-stim condition at 3-year follow-up (postoperative axial subscore off-med off-stim 8.9 ± 4.3 versus postoperative axial subscore off-med on-stim 6.8 ± 4.8, *P* = 0.04). After 3 years from DBS, dopaminergic drugs were significantly reduced by 31.7% (mean preoperative LEDD 1111.2 ± 505.9 mg versus mean postoperative LEDD 758.8 ± 356.7 mg, *P* = 0.04).

### Safety of frameless STN-DBS

No serious adverse events (e.g., hemorrhage, infection, or infarction) occurred during surgery. During 3-year follow-up, there was only one device-related adverse event: malfunction of one lead contact. Among the stimulation-related adverse events, dysarthria was the most frequent motor side effect: it occurred in four cases. One patient developed blepharospasm and another apraxia of eyelid opening. One patient had a single seizure after switching on the stimulator. One patient developed dementia 2 years after DBS.

## Discussion

In this study, we report the longest clinical follow-up of PD patients undergone bilateral STN-DBS with frameless stereotaxy and single-track multipass MER.

DBS allowed the reduction of dopaminergic therapy (31.2% and 31.7% at 1- and 3-year follow-up, respectively), while off-med UPDRS III score was improved (30.1% and 11.1% at 1- and 3-year follow-up, respectively). It has to be highlighted that comparison between preoperative and postoperative off-med UPDRS III scores at 3-year follow-up failed to reach significant difference either from a statistical or clinical point of view. This is a relevant observation; in fact, in other long-term follow-up studies, the UPDRS III improvement was sustained at least up to 5 years after DBS [2, 22]. While in these studies the patients had a high baseline UPDRS III score (ranging from 50.2 to 59.5), that remained substantially stable during the follow-up, in our study, the baseline UPDRS III score was lower and it worsened after 3 years (from 29.8 ± 10.2 to 41.6 ± 14.1). The different progression of motor scores among these populations might be explained by different severity of disease at baseline, being PD clinical progression rate higher in milder than in advanced stages [23]. Thus, selecting for DBS patients with milder motor impairment, in line with recent evidences [24], we found a worsening of baseline motor score after 3 years, as expected [23]. Therefore, considering the underlying disease progression, we compared postoperative off-med on-stim and postoperative off-med off-stim UPDRS III score at 3-year follow-up, founding a significant DBS motor efficacy of 36.3%.

Although the beneficial effects of STN-DBS on appendicular motor symptoms are well recognized, the response of axial disability to this intervention is more difficult to predict [25]. We specifically evaluated axial symptoms with the axial subscore of UPDRS III: an improvement of 36.4% was found at 1-year follow-up; at 3-year follow-up, the axial subscore was impaired of 11.5% compared with preoperative off-med condition, but it was significantly improved of 23.6% compared with postoperative off-med off-stim condition, probably because of the underlying disease progression (axial subscore impairment from 6.1 ± 3.0 preoperatively to 8.9 ± 4.3 postoperatively).

Our data at 1- and 3-year follow-up are in line with frame-based stereotaxy studies. For the large randomized-controlled studies with frame-based stereotaxy, at 6–12 months of follow-up, the reduction of off-med UPDRS III varied between 28.6 and 41%, and the levodopa equivalent dosage was reduced by 23–50% [1, 24, 26–28]. In long-term follow-up studies with frame-based stereotaxy, these benefits were sustained at 3-year follow-up, similar to our study [2, 22].

In this study, we achieved excellent results in terms of safety, as we did not have major adverse events surgery-related. No infection, suicide attempt, or death occurred during the 3-year follow-up. Only minor stimulation-related side effects (dysarthria, blepharospasm, apraxia of eyelid opening, seizures) and one device-related side effect (malfunction of one lead contact) were reported in long-term follow-up. One patient, a 61-year-old man with a multi-domain mild cognitive impairment before the intervention, developed dementia 2 years after the intervention. The low rate of major adverse events with frame-based stereotaxy calls for precaution when comparing these studies with ours, for the low number of patients included [29]. However, in terms of safety, frameless technique seems not to increase the risk of adverse events, compared with frame-based stereotaxy.

Therefore, frameless DBS has clinical efficacy and safety as comparable with frame-based stereotaxy, with several advantages for clinicians and patients. This approach can reduce patients’ discomfort during the intervention, allowing them to move and adjust their position, with less muscular tension and pain, and better tolerance for the procedure. Moreover, the application of fiducial markers prior to surgery, avoiding a preoperative imaging after that the frame has been fixed, makes surgery time shorter than with conventional frame, sparing valuable operative room resources and time, and allowing patients to spend less time in the uncomfortable off-medication state [12, 16]. Further advantages of this technique, as compared with the frame-based one, are the easier intraoperative examination of the patient and eventual access to the airway, the possibility to make minor adjustment in multiple planes to the entry point without adjustment of a frame and coordinates, and the absence of periodic recalibration of frame and arc, necessary with frame-based stereotaxy. On the other hand, potential disadvantages of frameless procedure are a more limited access and visualization of the bur hole, compared with frame-based approach, and the need of procedural experience, which has been shown to be necessary to improve the targeting accuracy [9].

The accuracy plays a key role in DBS efficacy, and has been investigated in several studies. It depends on several factors: brain shift during the surgery, procedural experience, technical errors in lead placement, intrinsic stability of the platform, CT-MRI imaging fusion errors, and fiducial localization discrepancies [9]. On this latter issue, a prospective study has demonstrated that repeated CT imaging of stationary bone fiducials resulted in a localization discrepancy of 0.7 mm on average [30]. However, while for bone fiducials the accuracy is in an acceptable submillimeter range, skin fiducial markers or surface tracing, used in other frameless systems, do not provide sufficient accuracy for DBS surgery [31]. Finally, with broad procedural experience, frameless technique has equivalent lead placing accuracy to frame-based stereotaxy. It is in a range of 1 to 3 mm on average for both the procedures [10–14].

In the last years, other surgical strategies have been developed to improve DBS accuracy. MRI-guided DBS, with intraoperative imaging control, allows to avoid frame-based co-registration and image fusion, and MRI relies on an anatomical target under real-time guidance, taking into account the brain shift during the procedure. MRI-guided approach showed a good accuracy, in average within a range of 0.6 to 2 mm, reducing to submillimeter error in most cases [32–34]. Moreover, robotic-assisted techniques have recently emerged to improve accuracy and precision of DBS lead placement. Interestingly, these procedures showed submillimeter accuracy using both frame-based and frameless systems [35–37], and demonstrated greater accuracy when compared with classic manual procedures [38, 39]. Future large studies should evaluate if MRI-guided and/or robotic-assisted techniques may further improve clinical DBS outcomes, compared with classic manual procedures.

This study has several limitations: the main one is the lack of a control group. In our center, we use only frameless technique, so, being this a single-center retrospective study, we do not have an available control group of patients operated with frame-based technique. The second limit of the study is the small size of the sample, which limits the statistical significance of the results. The third limit is the retrospective design of the study. The forth limit is the lack of targeting accuracy data, not available because of the retrospective nature of the study. Future large prospective controlled studies would be advisable to strengthen our findings.

## Conclusions

Bilateral STN-DBS in PD patients with frameless stereotaxy and single-track multipass MER results in good motor outcomes with an excellent safety profile at 1- and 3-year follow-up. The ranges of UPDRS III improvement and dopaminergic therapy reduction were as those reported using frame-based stereotaxy. No major adverse events during surgery were reported. In conclusion, frameless stereotaxy is an effective and safe technique for DBS surgery, with great advantages for patients’ discomfort during surgery.

## Abbreviations

DBS: Deep brain stimulation
PD: Parkinson’s disease
STN: Subthalamic nucleus
LEDD: Levodopa equivalent daily dose
